# Challenges for hand hygiene in a private hospital multi-cultural setting, Saudi Arabia

**DOI:** 10.1186/1753-6561-5-S6-P129

**Published:** 2011-06-29

**Authors:** RV Boychuk

**Affiliations:** 1 Infection Control, Saad Specialist Hospital, Al Khobar, Saudi Arabia

## Introduction / objectives

The awareness of hand hygiene in a complex medical setting has been historically emphasized; however, more recently, hand hygiene has been strongly linked with healthcare associated infections to the point where insurance companies have declined coverage in many countries.

## Methods

In Saad Specialist Hospital, we have been strongly proactive since the facility opened it's doors in 2001. Following the publications has been a challenge to our Infection Control Team and Committee. Supported by the IPSG and IHI has shown hand hygiene plays a major role in HAIs and has stimulated the IC team to adopt the detailed process of "5 Moments of Hand Hygiene" and incorporate the recommendations of WHO into all disciplines throughout the facility. We have assessed products, the location of sanitizers, and installing sanitizers at the "point of care", including the OP Clinics.

## Results

Intense teaching has been ongoing for over one year, however, it is difficult to attain and sustain our overall IHI goal of greater than 90% compliance. We have looked at different methods to increase awareness including giving certificates to units who have achieved over 90% for 3 or more months. At the end of each year, we have awarded a trophy to the highest scored unit sustaining the goal. We have called this the "Semmelwis Award", hence paying tribute to a great pioneer that has led us by hand to a healthier healthcare setting.

## Conclusion

By showing the changes that have taken over the past year, utilizing unit staff to audit their own units, has increased the awareness and compliance. We can show that consistent hand hygiene has definitely decreased the healthcare associated infections.

## Disclosure of interest

None declared.

